# Benefits of Creatine Supplementation for Vegetarians Compared to Omnivorous Athletes: A Systematic Review

**DOI:** 10.3390/ijerph17093041

**Published:** 2020-04-27

**Authors:** Mojtaba Kaviani, Keely Shaw, Philip D. Chilibeck

**Affiliations:** 1 School of Nutrition and Dietetics, Faculty of Pure & Applied Science, Acadia University, Wolfville, NB B4P 2R6, Canada; 2 College of Kinesiology, University of Saskatchewan, 87 Campus Dr, Saskatoon, SK S7N 5B2, Canada; keely.shaw@usask.ca

**Keywords:** sport performance, recovery, health, vegan

## Abstract

Background: Creatine monohydrate is a nutritional supplement often consumed by athletes in anaerobic sports. Creatine is naturally found in most meat products; therefore, vegetarians have reduced creatine stores and may benefit from supplementation. Objective: to determine the effects of creatine supplementation on vegetarians. Data sources: PubMed and SPORTDiscus. Eligibility criteria: Randomized controlled trials (parallel group, cross-over studies) or prospective studies. Participants: Vegetarians. Intervention: Creatine supplementation. Study appraisal and synthesis: A total of 64 records were identified, and eleven full-text articles (covering nine studies) were included in this systematic review. Results: Creatine supplementation in vegetarians increased total creatine, creatine, and phosphocreatine concentrations in vastus lateralis and gastrocnemius muscle, plasma, and red blood cells, often to levels greater than omnivores. Creatine supplementation had no effect on brain levels of phosphocreatine. Creatine supplementation increased lean tissue mass, type II fiber area, insulin-like growth factor-1, muscular strength, muscular endurance, Wingate mean power output, and brain function (memory and intelligence) in vegetarian participants. Studies were mixed on whether creatine supplementation improved exercise performance in vegetarians to a greater extent compared to omnivores. Limitations: Studies that were reviewed had moderate–high risk of bias. Conclusions: Overall, it appears vegetarian athletes are likely to benefit from creatine supplementation.

## 1. Introduction

### 1.1. The Benefits of Vegetarian Diets on Health and Performance

There are many reasons why an individual may choose to practice a vegetarian diet, including religious beliefs, ethical considerations, perceived health benefits, or philosophical beliefs [1]. Along the continuum of vegetarian diets, there are several plant-based options of varying restrictiveness. Closest to the standard omnivore diet are the semi-vegetarians who avoid some animal products, most commonly red meats. Lacto–ovo vegetarians avoid eating animals and only consume dairy products and eggs in addition to plant-based foods. Individuals can be further classified into ovo-vegetarians, only consuming plant-based foods and eggs, or lacto-vegetarians, only consuming plant-based foods and dairy products [1,2]. Finally, vegans exclude all animal products including dairy products, eggs, meats, seafood, gelatin, and honey [1]. 

There has been a sharp rise in athletes who consider a vegetarian diet due to the potential health benefits [2]. Diets including unrefined plant foods are positively correlated with overall health, lifespan, immune function, and cardiovascular health [3,4,5]. High-level athletes often show symptoms of upper respiratory tract infections due to long-term stress placed on the body [6]; thus, maximizing immune function is of great interest to this population. Excess amounts of fat and poor food choices increase exercise-induced suppression of the immune system. When done correctly, vegetarian diets offer the correct amount of healthy fats that will not only decrease immunosuppression but also increase immunocompetence [6,7]. In this light, the American Dietetic Association and Dietitians of Canada state that vegetarian diets are a healthy alternative for athletes of all ages [8]. On the other hand, a vegetarian diet could be lacking in certain macro- and micronutrients if they are not properly supplemented to dietary intake [2]. For instance, some vegetarians may experience low iron, vitamin B12, vitamin D, riboflavin, calcium, and zinc intakes as these nutrients are found in high quantities in animal products [2]. 

As with all high-performance athletes, basic dietary requirements must be met as well as health and performance needs in order to ensure optimal performance [1,2]. Vegetarians, especially vegans, often consume less energy than omnivores, largely because of the high consumption of fiber in vegetarian diets which promotes satiety [9,10,11]. A vegetarian diet may be lower in protein when compared to an omnivorous diet; therefore, a vegetarian diet requires careful planning to ensure adequate protein consumption [12,13]. Nutrition professionals must pay careful attention to the quantity and quality of protein that vegetarians are consuming because plant-based protein is often not a complete protein [13]. This means the protein is missing essential amino acids, including leucine, valine, phenylalanine, histidine, lysine, methionine, isoleucine, threonine, and tryptophan [14]. Of concern, methionine is a precursor of creatine [15], a natural compound with proven benefits on sport performance. 

### 1.2. Creatine

Creatine is naturally found in animal tissues such as meats, fish, and poultry. As a result, there is decreased intake among vegetarian athletes [2]. Dietary intake of creatine is significantly reduced in individuals following a vegetarian diet (egg and dairy products may contribute to provide very small amounts of creatine), while in vegan diets, almost no exogenous source of creatine is consumed [15]. The amount of creatine is lower in serum, plasma, red blood cells, and muscle, but not in the brain in vegetarians versus omnivores [16,17,18,19]. Further, vitamin B12 deficiency (common in vegetarians) is linked with impairment in methionine production, and this may lead to lower creatine biosynthesis [20]. Therefore, creatine supplementation appears to be a logical approach to offset some of the nutritional concerns associated with vegetarian diets [21]. 

### 1.3. Purpose of Creatine in Anaerobic Energy System Pathways

Approximately 1 g/day of creatine is synthesized by the body from arginine, glycine, and methionine [1]. In individuals consuming meat products, approximately 1 g/day is also consumed through foods. In vegetarian athletes, creatine stores could be lower because of reduced intake of meat. This has the possibility to affect their performance in high-intensity, short-term activities that largely rely on the anaerobic energy system pathways [1]. Approximately 90% of the body’s creatine is stored in muscle as free creatine and phosphocreatine [22]. During high-intensity exercise, phosphocreatine is broken down into creatine and a phosphate molecule. The phosphate molecule is then quickly paired with an ADP molecule to form ATP. This ATP is then used to power muscle contraction [22]. The concentration of phosphocreatine decreases rapidly after the onset of activity, leading to fatigue [12]. Starting an activity with a high store of phosphocreatine helps to enhance performance as well as recovery [22]. As vegetarians do not consume animal products, they may receive strong performance and recovery benefits with creatine supplementation.

## 2. How Does Creatine Supplementation Affect Omnivores?

Thousands of research trials have been conducted on the effects of creatine supplementation, mostly in omnivores; the results of these trials are summarized across a number of meta-analyses and systematic reviews [23,24,25,26,27,28,29]. The majority of data provide strong support indicating creatine supplementation is beneficial, in particular, for increasing upper and lower body strength [27,28,29], high-intensity anaerobic activities (especially when done in a repeated, intermittent fashion [23,25]), muscle mass [23,25,27], and recovery from exercise [25]; however, creatine supplementation is unlikely to improve aerobic exercise performance [24]. When combined with resistance training, creatine supplementation enables athletes to train at a higher intensity, leading to greater training adaptations, which result in greater anabolic gains [18,30]. During high-intensity and short-term exercise which relies mainly on anaerobic energy system pathways, it may be advantageous to have a larger creatine pool to increase anaerobic exercise capacity and shorten recovery time between bouts of exercise [25]. 

## 3. Differences in Creatine Levels in Muscle, Brain, and Blood in Vegetarians Versus Omnivores

Creatine is mainly derived from beef, fish, pork, and chicken [31,32]; therefore, vegetarians have lower dietary intakes [33] and potentially lower levels in blood, muscle, and other tissues, such as brain. In this section, we have considered differences between vegetarians and omnivores for creatine concentration in blood, muscle, and brain because blood gives a general reflection of whole-body creatine status, and muscle and brain are important organs with respect to exercise (i.e., considering that activation of the motor cortex is important for recruitment of motor neurons and muscle contraction).

Creatine or phosphocreatine concentrations are most easily measured in the blood, but they have also been measured in muscle by obtaining biopsy samples or using ^31^P-magnetic resonance spectroscopy (^31^P-MRS) and in brain by ^31^P-MRS or ^1^H-MRS. Creatine concentrations are lower by about 50% in plasma [34,35], by 35–39% in serum [16], and by 27–50% in red blood cells [16,36] in vegetarians compared to omnivores. 

Muscle biopsies from the vastus lateralis indicate that total creatine (i.e., creatine + phosphocreatine), phosphocreatine, and creatine concentrations are lower by 10–15%, 7–10%, and 7–26%, respectively, in vegetarians compared to omnivores [17,18,34,37]. In the lateral gastrocnemius, phosphocreatine levels, as assessed by ^31^P-MRS, are similar in vegetarians and omnivores (i.e., a reduction in vegetarians by ~5% that was not statistically significant) [33]; therefore, differences between vegetarians and omnivores seem to be muscle-specific. The similar muscle phosphocreatine levels in the gastrocnemius of vegetarians in this study do not seem to be related to duration of vegetarianism, since the vegetarians in this study had been vegetarians for about 10 years duration, which is longer than most studies. The gastrocnemius has more efficient phosphocreatine metabolism in response to exercise (i.e., a faster phosphocreatine initial rate of recovery following exercise) compared to the vastus lateralis [38]. Perhaps this greater efficiency allows better preservation of phosphocreatine status in conditions where dietary creatine intake is compromised (e.g., in vegetarians).

Unlike blood and muscle, creatine concentrations in the brain appear similar between vegetarians and omnivores; however, this has only been assessed in two studies. Using ^31^P-MRS, Solis et al. [33] found no differences for brain phosphocreatine concentrations, and using ^1^H-MRS, Solis et al. [19] found no differences for brain total creatine concentrations between vegetarians and omnivores. In summary, research to date indicates that creatine or phosphocreatine concentrations are lower in plasma, serum, red blood cells, and the vastus lateralis, but not in the lateral gastrocnemius or brain in vegetarians compared to omnivores.

## 4. Systematic Review of Creatine Supplementation in Vegetarians

Since creatine and phosphocreatine concentrations may be lower in the muscle of vegetarians, there is good potential that supplementation with creatine might improve creatine and phosphocreatine concentrations, and potentially enhance exercise performance and recovery in vegetarians. It should be noted that although creatine is found mostly in animal products, the creatine in most supplements is synthesized from sarcosine and cyanamide [39,40], does not contain any animal by-products, and is therefore “vegan-friendly”. The only precaution is that vegans should avoid creatine supplements delivered in capsule form because the capsules are often derived from gelatin and therefore could contain animal by-products. In our review of the literature, we found one systematic review on the effect of creatine supplementation in vegetarians [41]; however, it included a review of only three manuscripts with two of the manuscripts derived from the same study. The conclusions from this systematic review were therefore limited. Our objective was to systematically review randomized and prospective parallel groups or cross-over studies on the effect of creatine supplementation (compared to placebo) in vegetarians (compared to omnivores where possible) for outcomes including body creatine stores and exercise performance. We hypothesized creatine supplementation would increase creatine concentrations in the blood and muscle of vegetarians (to a greater extent than omnivores) and this would lead to improvements in anaerobic exercise performance. 

### 4.1. Systematic Review Methodology

Our systematic review was performed in accordance with the guidelines for the Preferred Reporting Items for Systematic Review and Meta-Analysis (PRISMA) statement. The literature search was conducted in PubMed and SPORTDiscus, including all dates up until 18 April 2020. Key words and Boolean phrases searched included “creatine” AND “(vegetarian OR vegan OR vegetarianism OR veganism)”. There were no restrictions on language or date. Since creatine supplementation can have short-term effects on muscle function, we considered any length of follow up. 

The following population, intervention, comparator, outcomes, and study types (PICOS) were included: The population was vegetarians. The intervention was creatine monohydrate supplementation. The comparator was either a placebo and/or participants who were omnivores. Outcomes included muscle, blood, or brain measurements of creatine or phosphocreatine, any exercise performance measure, lean tissue mass, muscle fiber area, anabolic hormones, creatine transporter levels, and cognitive performance. Study type included randomized controlled trials (parallel group and cross-over trials) and prospective trials.

Titles, abstracts, and full manuscripts were reviewed for inclusion by two reviewers. Data extraction and determination of risk of bias using the most recently revised Cochrane risk of bias tool [42] was also performed by two reviewers. When there were disagreements, the reviewers either came to a consensus or consulted a third reviewer.

### 4.2. Systematic Review Results

**Figure 1:**
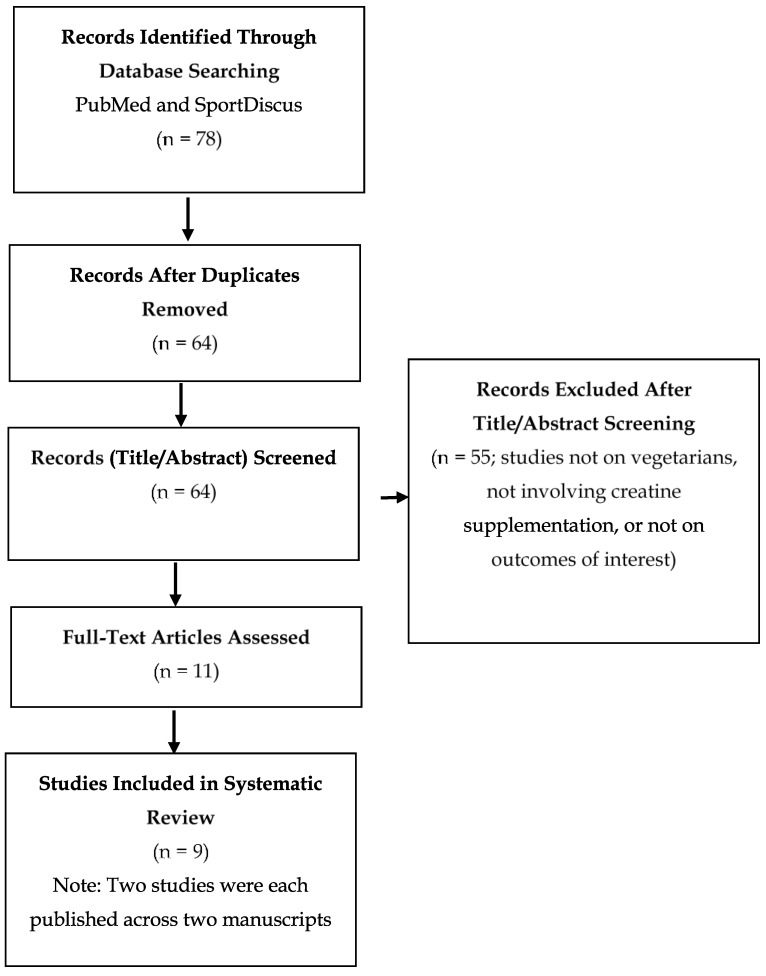
PRISMA diagram: Flow chart of study section process.

Figure 1 outlines the flow chart for the study selection process. Nine studies, published across 11 journal articles, were included. Creatine supplementation studies in vegetarians and effects on creatine concentrations and function of muscle and brain are summarized in Table 1. In Table 1, we present details such as type of vegetarian (i.e., vegan, lacto–ovo, ovo-vegetarianism) and length of vegetarianism of research participants. This was not always reported in the studies we reviewed; therefore, this is one aspect that can be improved in future studies, as these factors could impact response to creatine supplementation. Table 2 presents our risk of bias assessment of included studies. The overall risks of bias for most studies were classified as “some concerns” or “high”, with only one study considered “low”.

### 4.3. Systematic Review Discussion

Creatine supplementation in vegetarians is effective for increasing creatine and phosphocreatine levels to an extent that vegetarians may achieve higher levels of creatine and phosphocreatine after supplementation, compared to omnivores (i.e., it appears that the lower baseline levels in vegetarians might allow for “super compensation” of creatine or phosphocreatine levels with supplementation; see Table 1). For example, five to seven days of creatine supplementation (at a dose of about 20–25 g/day) results in greater increases in plasma creatine [36], vastus lateralis total creatine [37], and gastrocnemius phosphocreatine [33] concentrations in vegetarians versus omnivores, resulting in greater concentrations of creatine or phosphocreatine post-supplementation in vegetarians despite lower baseline levels. This leads to speculation that omnivores might be able to deplete their muscles of creatine (through meat abstinence) to achieve a super compensation of creatine and phosphocreatine levels with supplementation. Only one study has attempted this type of intervention. Lukaszuk et al. [17,43] randomized 26 male omnivores to receive 26 days of their regular diet or a lacto–ovo vegetarian diet, and further randomized these participants to 0.3 g creatine/kg/day supplementation or placebo in the last five days (i.e., days 22–26). During these last five days, total creatine in the vastus lateralis increased in the vegetarian creatine-supplemented group by 20% and the omnivorous creatine-supplemented group by 10% [17]. This was significantly greater than the placebo-supplemented groups, but because of low participant numbers and therefore lack of statistical power, the increases between the vegetarian-supplemented group and omnivorous-supplemented group were not statistically significant. Phosphocreatine concentrations were 22% higher at the end of the five days of creatine supplementation in the vegetarian compared to the omnivorous group, but again this was not statistically different, most likely due to low statistical power. One would expect, however, that a 22% difference in muscle phosphocreatine would lead to performance differences during short-term, high-intensity exercise, but this was not assessed in this study. This is an area open for future research to inform athletes involved in anaerobic sports: whether depleting the muscle of creatine (i.e., through adoption of a vegetarian diet) followed by creatine supplementation might lead to greater enhancements in sport performance compared to just supplementation alone. 

With an apparently enhanced ability to take up and store creatine, one might expect the muscle creatine transporter to be enhanced with a vegetarian diet. This was assessed in one study where seven male vegetarians (with at least six months on a vegetarian diet) and seven male omnivores participated in a randomized cross-over study involving 0.4 g creatine/kg/day or placebo for five days [37]. Baseline mRNA levels for the creatine transporter from biopsies of the vastus lateralis were not different between vegetarian and omnivore groups; they increased after one day of creatine supplementation (in vegetarian and omnivore groups combined) and then returned to baseline by day 5. Changes in mRNA levels do not always result in increased protein expression because further steps (i.e., translation) need to be activated for full protein synthesis. It would be of interest for future studies to assess protein levels of the creatine transporter in vegetarian versus omnivorous individuals.

The minimum level of creatine supplementation required in vegetarians to prevent decreased creatine stores is probably about 1 g/day (the amount found in 200 g of steak) [47]. Blancquaert et al. [34] randomized 40 female omnivores, with 10 continuing their normal diet, 15 going on a lacto–ovo vegetarian diet and supplementing with 1 g creatine/day, and 15 going on a lacto–ovo vegetarian diet and supplementing with placebo for six months. Vastus lateralis biopsies were collected at baseline and three months, and plasma at baseline, three months, and six months. After three months, total creatine in the vastus lateralis decreased 15% in the vegetarian + placebo group, and increased 9.7% in the vegetarian + creatine group and 6.8% in the control group. After six months, plasma creatine had decreased 46% in the vegetarian + placebo group, increased 195% in the vegetarian + creatine group, and did not change in the control group, compared to baseline. This indicates that just a small amount of creatine supplementation (i.e., 1 g/day) is required for vegetarians to prevent reductions in muscle creatine levels.

Since vegetarians respond better than omnivores to creatine supplementation, it might be expected that creatine would enhance exercise performance to a greater extent in vegetarians; however, studies are mixed (Table 1). The study by Blancquaert et al. [34] discussed in the previous paragraph assessed exercise performance during an incremental cycle ergometer test over six months in groups assigned to vegetarian diet, omnivorous diet, and vegetarian diet + 1 g creatine/day, but despite differences between groups for creatine levels in plasma and muscle, no differences were observed between groups for exercise performance. The performance test used, however, is more of a test of aerobic capacity, rather than a test that would stress anaerobic energy systems and muscular phosphocreatine usage; therefore, lack of effectiveness of creatine supplementation on this test is not surprising [24]. Watt et al. [37] assessed changes in performance during two repeats of a 30 s Wingate test, separated by four minutes rest during a cross-over study of 0.4 g creatine/kg/day or placebo for five days in seven vegetarian and omnivorous males. This type of test involves short-duration maximal cycling, largely depends on the usage of muscle phosphocreatine stores, and improves with creatine supplementation in studies of omnivorous participants [24]. Despite a greater increase in vastus lateralis total creatine in vegetarians, both the vegetarians and omnivores equally increased mean power output during the second Wingate test with creatine supplementation. This could be due to a roughly equivalent (~31%) increase in vastus lateralis phosphocreatine in both groups (i.e., the greater total creatine in the vegetarian group would be due to greater increase in free creatine, whereas it is the phosphorylated creatine that is essential for re-phosphorylating adenosine diphosphate to re-synthesize adenosine triphosphate necessary for repeated high-force muscle contractions). A limitation discussed by Watt et al. [37] is that the washout period of five weeks between creatine and placebo phases may not have been sufficient for the vegetarian participants. Of the three vegetarian participants who received creatine supplementation during the first phase of the cross-over, their vastus lateralis total creatine levels were still elevated at the start of their placebo phase. This lack of sufficient washout of muscle creatine may have artificially enhanced performance during the placebo phase, therefore, attenuating any performance difference between creatine and placebo phases in the vegetarian participants. In contrast to what might be expected with vegetarians being more responsive to creatine supplementation, Shomrat et al. [35] found that omnivores might have slightly better improvement in anaerobic performance than vegetarians with creatine supplementation. In their study, seven male vegetarians and nine omnivores received 3 × 7 g creatine/day for six days. Mean power output during 3 × 20 s Wingate cycle tests (with four-minute recovery between bouts) was increased in both supplemented groups by about 5%, but only the omnivores had an increase in peak power output during the test (by about 5%). 

A limitation of the creatine supplement studies in vegetarians presented Table 1 is that most assessed non-athletic populations. Burke et al. [18,44] were the only researchers to assess recreational athletes, who either walked, jogged, swam, or cycled on a regular basis, and also had one to five years of resistance training experience. This study randomized 18 male and female vegetarians and 24 male and female omnivores to creatine (0.25 g creatine/kg lean tissue mass/day for seven days + 0.0625 g creatine/kg lean tissue mass/day for 56 days) or placebo groups. During supplementation, they engaged in a high-volume resistance training program (involving a three-day split which trained all major muscle groups over the three days). Vegetarians on creatine had a greater increase in vastus lateralis total creatine and phosphocreatine than other groups during the intervention (Table 1). This translated to a greater increase in lean tissue mass and greater ability to perform work during a muscular endurance task involving 50 repetitions of maximal knee extension/flexion exercise on an isokinetic dynamometer at a velocity of 180 degrees/s [18]. The difference in this study compared to other studies for enhancement of exercise performance preferentially in vegetarians who supplemented with creatine could be due to the longer-term supplementation, the use of an athletic population (who would have less variation from day to day on exercise performance tests than a non-athletic population), and that creatine supplementation was combined with a resistance training program. The athletes in the Burke et al. [18,44] study were described as “recreational” athletes. An area for future research would be to assess the effect of creatine supplementation on a more elite group of vegetarian athletes.

Although most creatine in the body is stored in muscle, the brain is also dependent on phosphocreatine as an energy source and therefore there is interest in the effects of creatine supplementation on brain function. Brain function is important for athletes because activation of the motor cortex is essential for recruiting spinal motor neurons and contraction of muscle fibers [48]. As mentioned previously, it appears that unlike skeletal muscle, creatine and phosphocreatine levels in the brains of vegetarians are not lower compared to omnivores [19,33]. Creatine supplementation (0.3 g/kg/day for seven days) in vegetarians and omnivores failed to increase phosphocreatine levels in the brain [33]. One might therefore expect equal responsiveness to creatine supplementation in vegetarians and omnivores for effects on brain function, but this is not the case (Table 1). Benton et al. [45] randomized 70 female vegetarians and 51 omnivores to 20 g creatine/day or placebo for five days and found that memory was enhanced in vegetarians supplemented with creatine but not omnivores. Rae et al. [46] supplemented 45 vegetarians with 5 g creatine/day or placebo for six weeks in a cross-over study and found working memory and intelligence were increased with creatine supplementation compared with placebo. These studies therefore show that memory and intelligence can be enhanced in vegetarians with creatine supplementation; this may translate to better sport performance (for example, a competitive vegetarian athlete on creatine might be better able to remember strategies used by an opponent during past competitions). This, however, remains to be tested in an athletic situation. It would be of interest to determine if creatine supplementation enhances the ability to activate the motor cortex during exercise to recruit motor units. This has been a mechanism proposed to explain the enhancement of exercise responses with creatine supplementation in omnivorous participants [49], but has never been directly tested. 

## 5. Conclusions

Taken together, creatine supplementation has the ability to increase performance in vegetarians as well as omnivores; however, the research is not conclusive on whether vegetarians show a greater increase in performance than their omnivore peers. Most of the studies we reviewed were limited by moderate to high risks of bias (Table 2); therefore, future studies of creatine supplementation in vegetarians could be improved by more vigorous randomized controlled trial designs. 

There are a number of factors that may influence the effectiveness of creatine supplementation such as the amount of total stored creatine and how long the individual has been a vegetarian. Creatine supplementation could be useful, particularly in stricter vegetarians such as vegans, since their total creatine stores will be lower, and as a result, they will see the greatest increases in creatine stores with supplementation. Additionally, it would be useful in individuals who have been vegetarians for a while, longer than six weeks, as this is has been shown to be the washout time for creatine supplementation for vegetarians. Overall, creatine supplementation could be useful for any athletes who have low pre-existing muscle creatine stores, and this is typical in vegetarians. Further research is still necessary to see how creatine supplementation affects elite-level vegetarian athletes, and the different types of vegetarians. 

## Figures and Tables

**Table 1:** Studies involving creatine supplementation in vegetarians.

Study	Participants	Design	Results
Solis et al. [33]	8 male, 6 female vegetarians (4 vegan, 9 lacto–ovo, 1 ovo); duration vegetarian ~10 y 11 male, 6 female omnivoresMean age ~30 y Approximately equal number in each group classified as having low, moderate, and high levels of physical activity	Non-randomized cross-over study Participants were given placebo for 7 d, then 0.3 g Cr/kg/d for 7 d	Gastrocnemius PCr increased by 25% after Cr supplementation in vegetarians with no increase in omnivores. Gastrocnemius PCr was about 5% lower before Cr supplementation in vegetarians but 17% higher after Cr supplementation versus omnivores. Brain PCr: no change in either group with Cr supplementation.
Blancquaert et al. [34]	40 female omnivores Mean age ~26 y	RCT n = 10 controls n = 15 given a lacto–ovo vegetarian diet for 6 months + 1 g Cr/d n = 15 given lacto–ovo vegetarian diet for 6 months + placebo	After 3 months: Vastus lateralis TCr decreased 15% with vegetarian + placebo; increased 9.7% with vegetarian + Cr; increased 6.8% with control. After 6 months: Plasma Cr decreased 46% with vegetarian + placebo; increased 195% with vegetarian + Cr; no change with control. Performance on an incremental cycling test to exhaustion: no change. over the 6 months
Shomrat et al. [35]	7 male vegetarians 17 male omnivores Active, but not highly trained Mean age ~28 y	Non-randomized prospective 9 omnivores and the 7 vegetarians received 3 × 7 g Cr/d for 6 d 8 omnivores received placebo	Performance on 3 × 20 s modified Wingate maximal cycling tests with 4 min recovery between bouts: Mean power output increased 5% in vegetarians and omnivores supplemented with Cr, but not with placebo. Peak power output increased 5% only in omnivores on Cr.
MacCormick et al. [36]	6 female vegetarians 6 female omnivores Mean age ~22 y Physically active, but not athletes	Prospective Participants were given 0.3 g Cr/kg LTM/d for 5 d	Vegetarians increased Cr in erythrocytes (140%) and plasma (258%); omnivores increased Cr in erythrocytes (53%) and plasma (116%). At 5 d, vegetarians had 89% higher plasma Cr compared to omnivores.
Watt et al. [37]	7 male vegetarians (4 vegans, 3 lacto–ovo vegetarians), at least 6 months duration vegetarian; mean age 23 y 7 male omnivores, mean age 28 y	Randomized cross-over study 0.4 g Cr/kg/d or placebo for 5 d 5-week washout	Vastus lateralis TCr increased 76% in vegetarians with Cr supplementation versus 35% in omnivores. Vastus lateralis PCr increased by ~31% in both groups with Cr supplementation. Vastus lateralis TCr was lower in vegetarians at baseline, but 12% higher than omnivores after 5 d Cr supplementation. Vastus lateralis Cr transporter mRNA increased 1 d into Cr supplementation in both groups, then returned to baseline levels at 5 d. 2 × 30 s Wingate maximal cycling test with 4 min recovery between bouts: Cr supplementation increased mean power output for both groups on the 2nd bout.
Lukaszuk et al. [17,43]	26 male omnivores Mean age 23.5 y Physically active, but not strength trained	RCT n = 14 controls n = 12 given lacto–ovo vegetarian diet for 26 dOn day 22: Participants were randomized to receive 0.3 g Cr/kg or placebo	After 21 d, participants who were given the lacto–ovo diet decreased vastus lateralis TCr (9.5%), PCr (8.7%), Cr (11%), and plasma Cr (9.1%). From day 22 to 27, TCr increased in the vegetarian Cr-supplemented group (20%) and the omnivorous Cr-supplemented group (10%) compared to the vegetarian placebo group (−2%) and the omnivorous placebo group (0%). Plasma Cr increased across Cr supplementation groups (13.3%) compared to placebo groups (0.5%).
Burke et al. [18,44]	8 male, 10 female vegetarians (3 vegans, 15 lacto–ovo vegetarians; vegetarian duration at least 3 y) 12 male, 12 female omnivores All participants were recreational athletes (walking, jogging, swimming, cycling) with 1–5 y resistance training experience Mean age ~33 y	RCT n = 5 male, 5 female vegetarians and 7 male, 5 female omnivores given 0.25 g Cr/kg LTM/d for 7 d, 0.0625 Cr/kg LTM/d for 49 d n = 3 male, 5 female vegetarians and 5 males, 7 female omnivores given placebo for 56 d All participants engaged in a high-volume resistance training program (training all major muscle groups) for 56 d	Vegetarians on Cr had greater increase in vastus lateralis PCr (+66%) than omnivores on Cr (+19%), vegetarians on placebo (−18%), and omnivores on placebo (+6%). Vegetarians on Cr had greater increase in vastus lateralis TCr (+30%) than omnivores on Cr (+8%), vegetarians on placebo (−4%), and omnivores on placebo (+4%). Vegetarians on Cr increased lean tissue mass 2.4 kg, which was greater than omnivores on Cr (1.9 kg) and other groups (1 kg). Vegetarians on Cr had greater increase in total work during 50 isokinetic knee extensions/flexions (30%) than omnivores on Cr (9%), vegetarians on placebo (8%), and omnivores on placebo (4%). Both creatine groups increased bench press strength, type II vastus lateralis fiber area, and muscle insulin-like growth factor-1 more than placebo groups.
Benton et al. [45]	70 female vegetarians 51 female omnivores Mean age ~20.3 y	RCT Participants given either 20 g Cr/d or placebo for 5 d	Memory was enhanced in vegetarians on Cr, but not omnivores.
Rae et al. [46]	45 vegetarians (12 males, 33 females; 18 vegans for median duration 4.6 y; 27 lacto–ovo vegetarians for median duration 14.3 y) Mean age ~26 y	Randomized cross-over study 6 weeks with 5 g Cr/d or placebo 6-week washout	Working memory and intelligence were increased during creatine compared to placebo supplementation.

Abbreviations: Cr = creatine; LTM = lean tissue mass; PCr = phosphocreatine; RCT = randomized controlled trial; TCr = total creatine.

**Table 2:** Risk of bias in studies involving creatine supplementation in vegetarians.

Study	Risk of Bias Domains					
	Randomization Process	Deviations from the Intended Intervention	Missing Outcome Data	Measurement of the Outcome	Selection of the Reported Result	Overall Risk of Bias
Benton et al. [45]	Some concerns	Low	Low	Low	Low	Some concerns
Blancquaert et al. [34]	Low	Low	Low	Low	Low	Low
Burke et al. [18,44]	Some concerns	Low	Low	Low	Low	Some concerns
Lukaszuk et al. [17,43]	Some concerns	Low	Low	Some concerns	Low	Some concerns
MacCormick et al. [36]	High	Some concerns	Low	Low	Low	High
Rae et al. [46]	High	Low	Low	Low	Low	High
Shomrat et al. [35]	High	Low	Low	Some concerns	Low	High
Solis et al. [33]	High	High	Some concerns	Low	Low	High
Watt et al. [37]	High	Some concerns	Some concerns	Low	Low	High
